# Knowledge, attitudes and practices towards antibiotic use in upper respiratory tract infections among patients seeking primary health care in Singapore

**DOI:** 10.1186/s12875-016-0547-3

**Published:** 2016-11-03

**Authors:** Darius Shaw Teng Pan, Joyce Huixin Huang, Magdalene Hui Min Lee, Yue Yu, Mark I-Cheng Chen, Ee Hui Goh, Lili Jiang, Joash Wen Chen Chong, Yee Sin Leo, Tau Hong Lee, Chia Siong Wong, Victor Weng Keong Loh, Adrian Zhongxian Poh, Tat Yean Tham, Wei Mon Wong, Fong Seng Lim

**Affiliations:** 1 Yong Loo Lin School of Medicine, National University Health System, National University of Singapore, 119228 Singapore, Singapore; 2 Saw Swee Hock School of Public Health, National University Health System, National University of Singapore, 12 Science Drive 2, 117549 Singapore, Singapore; 3 Institute of Infectious Diseases & Epidemiology, Communicable Disease Centre, Tan Tock Seng Hospital, 308433 Singapore, Singapore; 4 Lee Kong Chian School of Medicine, Nan Yang Technological University, 308232 Singapore, Singapore; 5 Division of Family Medicine, Department of Medicine, University Medicine Cluster, National University Hospital System, 119228 Singapore, Singapore; 6 Frontier Healthcare group, 400305 Singapore, Singapore; 7 Division of Primary Care, Raffles Medical Group, 188770 Singapore, Singapore; 8 Duke NUS Graduate Medical School, National University of Singapore, 169857 Singapore, Singapore

**Keywords:** Antibiotic use, Upper Respiratory Tract Infections (URTIs), Primary healthcare, Singapore, Educational level

## Abstract

**Background:**

Patients’ expectations can influence antibiotic prescription by primary healthcare physicians. We assessed knowledge, attitude and practices towards antibiotic use for upper respiratory tract infections (URTIs), and whether knowledge is associated with increased expectations for antibiotics among patients visiting primary healthcare services in Singapore.

**Methods:**

Data was collected through a cross-sectional interviewer-assisted survey of patients aged ≥21 years waiting to see primary healthcare practitioners for one or more symptoms suggestive of URTI (cough, sore throat, runny nose or blocked nose) for 7 days or less, covering the demographics, presenting symptoms, knowledge, attitudes, beliefs and practices of URTI and associated antibiotic use. Univariate and multivariate logistic regression was used to assess independent factors associated with patients’ expectations for antibiotics.

**Results:**

Nine hundred fourteen out of 987 eligible patients consulting 35 doctors were recruited from 24 private sector primary care clinics in Singapore. A third (307/907) expected antibiotics, of which a substantial proportion would ask the doctor for antibiotics (121/304, 40 %) and/or see another doctor (31/304, 10 %) if antibiotics were not prescribed. The majority agreed “antibiotics are effective against viruses” (715/914, 78 %) and that “antibiotics cure URTI faster” (594/912, 65 %). Inappropriate antibiotic practices include “keeping antibiotics stock at home” (125/913, 12 %), “taking leftover antibiotics” (114/913, 14 %) and giving antibiotics to family members (62/913, 7 %). On multivariate regression, the following factors were independently associated with wanting antibiotics (odds ratio; 95 % confidence interval): Malay ethnicity (1.67; 1.00–2.79), living in private housing (1.69; 1.13–2.51), presence of sore throat (1.50; 1.07–2.10) or fever (1.46; 1.01–2.12), perception that illness is serious (1.70; 1.27–2.27), belief that antibiotics cure URTI faster (5.35; 3.76–7.62) and not knowing URTI resolves on its own (2.18; 1.08–2.06), while post-secondary education (0.67; 0.48–0.94) was inversely associated. Those with lower educational levels were significantly more likely to have multiple misconceptions about antibiotics.

**Conclusion:**

Majority of patients seeking primary health care in Singapore are misinformed about the role of antibiotics in URTI. Agreeing with the statement that antibiotics cure URTI faster was most strongly associated with wanting antibiotics. Those with higher educational levels were less likely to want antibiotics, while those with lower educational levels more likely to have incorrect knowledge.

**Electronic supplementary material:**

The online version of this article (doi:10.1186/s12875-016-0547-3) contains supplementary material, which is available to authorized users.

## Background

The rise in antibiotic resistance has become an increasing public health concern worldwide [1]. In Singapore, patients admitted to local public hospitals have one of the highest rates of antimicrobial resistance worldwide [2, 3], with local use of oral antibiotics in the community shown to be associated with increased colonization with Extended-Spectrum Beta-Lactamase (ESBL) Gram-Negative Bacteria on admission [4]. The impact of antibiotic resistance include increased morbidity and mortality from antibiotic-resistant infections [5], increased socioeconomic burden and greater healthcare costs [1, 6].

Poor antibiotic stewardship is a key driver of antibiotic resistance [7]. A substantial proportion of all antibiotics are prescribed in the community [8], and Upper Respiratory Tract Infection (URTI) is one of the commonest conditions in the primary care setting for which antibiotic prescriptions have been reported to be high worldwide [9–11]. In Singapore, URTIs account for a quarter of all primary care attendances [12]. While there is no local data on antibiotic use for URTIs in Singapore, it has been noted that antibiotics are commonly prescribed for URTIs [13]. However, current evidence-based guidelines do not support antibiotic use in the majority of URTI cases [14, 15], as URTIs are frequently of viral etiology [16–18]. are often self-limiting [19, 20], and seldom lead to serious complications [21].

Inappropriate expectations of antibiotics by patients have been commonly observed in primary healthcare, and is a key factor driving over-prescription of antibiotics in such settings. For instance, Linder et al found that physicians are more likely to prescribe antibiotics to patients who desire antibiotics [22]. Furthermore, Scott et al observed that various inappropriate behaviours by patients often pressured physicians to prescribe antibiotics [23], such as direct request for antibiotics, portraying severity of illness, or volunteering previous positive experience with use of antibiotics. Lam et al also observed that primary healthcare physicians over-prescribe antibiotics in order to satisfy their patients [24]. These studies underscore how patient’s expectations for antibiotics influence prescriptions by physicians.

In Singapore, the majority of URTI patients (87 %) are seen in private sector general practitioner (GP) clinics [12]. We hence surveyed patients of GP clinics to identify possible strategies and specific areas for health education on better antibiotic stewardship. Specifically, our study aimed to describe the prevalence of misconceptions about URTIs and antibiotics, and identify key misconceptions associated with inappropriately wanting antibiotics. We also assessed if any misconceptions were especially prevalent in particular population subgroups.

## Methods

### Study design and setting

We conducted a cross-sectional study over eight working days in February 2015. Patients were recruited from those seeing 35 GPs at 24 clinics of various sizes, including both solo and group practices across Singapore in both residential and commercial areas. We were referred to these GPs mainly through the academic medicine network affiliated with the National University of Singapore. Following initial contact via emails or phone calls, site visits were conducted for interested GPs where the study objectives and execution were explained. Consent was obtained to survey patients at each clinic.

Fieldwork was conducted by a team of 38 fourth-year medical students from the National University of Singapore (NUS) Yong Loo Lin School of Medicine (YLLSoM) who were deployed in pairs to participating clinics during operating hours. These students underwent a carefully planned full day training program including video demonstrations, simulation and role-play to familiarize them with the study protocol and standardize the process of administering the questionnaire.

We aimed to study all patients aged 21 years and above, presenting with at least one of four URTI symptoms (runny nose, blocked nose, cough or sore throat) for seven days or less at participating clinics. Patients were excluded if they had sought medical consultation for the same symptoms in the preceding 30 days, were on long-term immunosuppressive or oral corticosteroid medications, had chronic kidney disease, had a past history of advanced stage or metastatic cancer, were immunocompromised (e.g. human immunodeficiency virus infection), or were not conversant in English or Mandarin. Eligible patients who provided written consent were enrolled into the study.

Following enrolment, researchers administered an interviewer-assisted pre-consultation questionnaire, in the clinics’ waiting rooms. The questionnaire was designed to include several factors identified from other studies, in particular a previous study done in Singapore [13]. During the design process, we engaged in consultations with a panel of experts including primary care physicians, infectious disease experts and public health experts to guide questionnaire development. The draft questionnaire was then piloted in a group of lay person volunteers before being field tested in a group of five clinics with actual patients to assess their understanding of individual questionnaire items. Inputs from these patients and their physicians were then used to refine the phrasing of the questionnaire. The questionnaire elicited details about the patients’ demographics, current episode of illness, knowledge, attitudes and beliefs about URTI and antibiotics, antibiotic practices and health-seeking behavior including wanting antibiotics. In order to elicit responses about URTI from lay participants, we referred to URTI as respiratory infection with common cough and flu symptoms in the questionnaire.

### Power calculations

Based on the available manpower resources and study timeframe, we projected recruitment of up to 1000 participants for this study. Assuming that approximately 40 % of patients would want antibiotics based on existing literature [22, 25], this gave an estimated margin of error of 3 % at 95 % confidence level in estimating the proportion of patients who expected antibiotics. It would also give us a power of 92 % to detect, at *p* < 0.05, factors that are at least 20 % more common in those who expected antibiotics as compared to those who did not.

### Data management and analysis

Data collected at each GP clinic was double-entered into a shared database. Frequency tabulations were performed for all descriptive data, with 95 % confidence intervals presented where relevant.

To facilitate interpretation, we dichotomised the responses to several survey items. For questions on attitudes, beliefs and practices, participants’s agreement to a given statement was measured on a 4-point scale from “strongly disagree” to “strongly agree” (e.g. “I believe that antibiotics cure my respiratory infection faster”). The participants who responded as “strongly agree” and “agree” were grouped as agreeing, while those who “strongly disagree” and “disagree” were considered as disagreeing with the statement. In survey items assessing knowledge, participants could either answer “yes”, “no” or “not sure” in response to a given statement where there was a designated correct answer (e.g. “Viruses cause most respiratory infections” where the correct answer is yes). Those who answered correctly were considered as giving the appropriate response, while those who replied as “not sure” or gave the incorrect answer were grouped as having an incorrect response.

We also investigated factors associated with whether a patient wanted antibiotics as a key outcome of interest, based on the response to the question “I want to receive antibiotics”. As above, those who responded as “strongly agree” and “agree” were considered as wanting antibiotics and vice-versa for “strongly disagree” and “disagree”. Logistic regression, with robust standard errors to account for potential clustering of results at the GP level, was performed to ascertain factors associated with wanting antibiotics, including participant’s sociodemographic factors (age, gender, ethnicity, employment, housing), episodic factors (symptom duration, presenting symptoms, payment mode), perception of illness severity, and questions pertaining to knowledge and beliefs about antibiotic use. Univariate and multivariate odds ratios (ORs) with 95 % confidence intervals (CIs) were calculated, with the multivariate estimates adjusting for all other covariates that were also assessed in univariate analysis. The exception was our decision to exclude responses reflecting practices (e.g. “I take leftover antibiotics when I have similar symptoms”), as these were statements describing behaviours which might arise from the same underlying motivations which make patients want antibiotics. However, we assessed the association between these inappropriate practices as well as with wanting antibiotics, separately presenting phi coefficients from Pearson’s correlation as a measure of association between these factors. Finally, we also assessed if particular beliefs or incorrect knowledge might be especially prevalent in particular sociodemographic subgroups, presenting *p*-values from chi-squared and Fisher’s exact tests.

All data was analyzed using Stata for Windows, version 11 (Stata Corporation, College Station, Texas, USA), with *p*-values of less than 0.05 considered statistically significant.

### Ethics approval

The study was approved by the institutional review board of the National University of Singapore (reference B-14-259).

## Results

Out of a total of 987 eligible patients, 914 patients gave signed informed consent to participate in the study (response rate = 92.6 %). Table 1 compares their socio-demographic profiles against 2014 population trends for Singapore [26]. The median age of participants was 35 years, and there were approximately equal numbers of males and females. Our study had similar proportions of each major ethnic group compared to that of the general Singapore residential population, though there were slightly less Chinese and more participants of other ethnic groups in our study. Compared to the general population, study participants were of a higher education level with a lower proportion having primary education and below, and a higher proportion with post-secondary education. The proportions staying in public and private housing were similar to that of the general population. Majority of our patients had partial or full subsidy of the payment for that visit from either pre-paid insurance or government subsidy schemes.

**Table 1:** Characteristics of study participants (*N* = 914) in comparison with 2014 Population Trends

Characteristic		Study participants (*N* = 914)	Singapore residents^a,d^
		No. (%)	(*N* = 3,870,739)
Age in years	Median	35.0	39.3
	21–34	438 (47.9)	821,864 (21.2)
	35–49	308 (33.7)	926,585 (23.9)
	50–64	121 (13.2)	835,397 (21.6)
	≥65	47 (5.1)	431,601 (11.2)
Gender	Male	443 (48.5)	1,900,513 (49.1)
	Female	471 (51.5)	1,970,186 (50.9)
Ethnicity	Chinese	630 (68.9)	2,874,380 (74.3)
	Malay	116 (12.7)	516,657 (13.3)
	Indian	99 (10.8)	353,021 (9.1)
	Other	69 (7.6)	126,681 (3.3)
Highest qualification attained^b^	Primary and below^c^	47 (5.1)	833,300 (31.2)
	Secondary	187 (20.5)	501,200 (18.8)
	Post-secondary	679 (74.4)	1334,700 (50.0)
Employment status	Currently employed	781 (85.4)	-
	Not currently employed	70 (7.7)	-
	Student	63 (6.9)	-
Housing type^b^	Public Housing	745 (81.7)	3154,691 (81.5)
	Private Housing	167 (18.3)	678,808 (17.5)
Mode of payment	Full Payment	275 (30.1)	-
	Partial Subsidy	385 (42.1)	-
	Full Subsidy	254 (27.8)	-

^a^Singaporeans and Singapore permanent residents only. Data taken from Singapore population trends 2014 unless specified

^b^Excludes 1 observation with missing data on highest qualification attained and 2 observations with missing data on housing type

^c^Equivalent to 6 years of formal education or less

^d^Department of Statistics Singapore. Population Trends 2014. Singapore: 2014

**Fig. 1:**
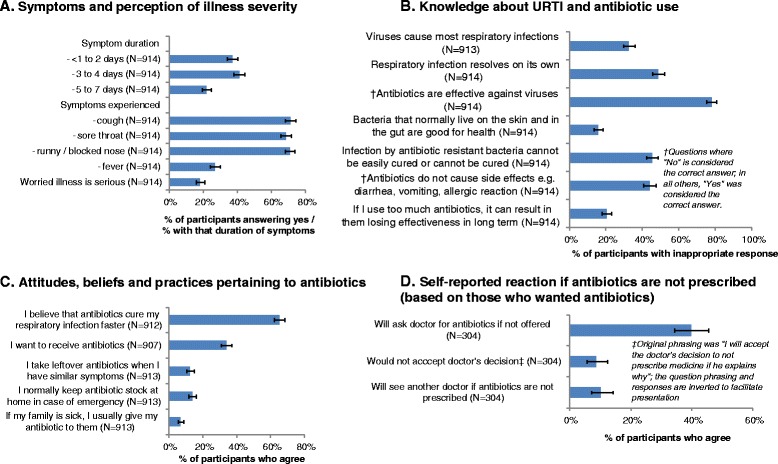
Prevalence of symptoms, knowledge, attitudes and practices among study population, which includes prevalence of **a** Type of symptom presentations and perception of illness severity, **b** Knowledge about URTI and antibiotic use, **c** Attitudes, beliefs and practices pertaining to antibiotics and **d** Self-reported response among patients who wanted antibiotics if antibiotics are not prescribed to them

Our patients presented to the clinics with mainly symptoms of cough, sore throat and runny or blocked nose, with duration of illness mostly between 1 to 4 days; 18.2 % of patients were worried that their illness was something serious (Fig. 1a). 32.6 % (298/913) did not know that viruses are the cause of most URTIs, but nearly half (48.8 %, 446/914) did not know URTI resolves on its own, and 78.3 % (715/914) did not reject the statement that antibiotics were effective against viruses (Fig. 1b). Also, nearly half (44.0 %, 402/914) did not know antibiotics have side effects, though the majority (79.5 %, 727/914) did at least know using antibiotics can result in lack of effectiveness in the long term. Close to two thirds (65.1 %, 594/912) agreed that antibiotics cure URTI faster, while a third (33.8 %, 307/907) wanted antibiotics (Fig. 1c), of which 39.8 % (121/304) would ask the doctor for antibiotics if not given; however, only 8.6 % (26/304) would not accept the doctor’s decision if the doctor explains why antibiotics were not prescribed, and 10.2 % (31/304) would see another doctor (Fig. 1d). Figure 1c also shows the prevalence of poor antibiotic practices, which include: “keeping antibiotic stock at home” (125/913, 13.7 %), “taking leftover antibiotics” (114/913, 12.4 %) and giving antibiotics to family members (62/913, 6.8 %). These practices were significantly correlated with each other at *p* < 0.001 (phi coefficients 0.40–0.50) and with wanting antibiotics (Table 2).

**Table 2:** Correlation between inappropriate practices and wanting to receive antibiotics for URTI

Inappropriate practices and wanting antibiotics	phi-coefficient (*p*-value)		
	I take leftover antibiotics when I have similar symptoms	I normally keep antibiotic stock at home in case of emergency	If my family is sick, I usually give my antibiotic to them
I normally keep antibiotic stock at home in case of emergency	0.495 (<0.001)	-	-
If my family is sick, I usually give my antibiotic to them	0.425 (<0.001)	0.399 (<0.001)	-
I want to receive antibiotics	0.172 (<0.001)	0.175 (<0.001)	0.130 (<0.001)

**Fig. 2:**
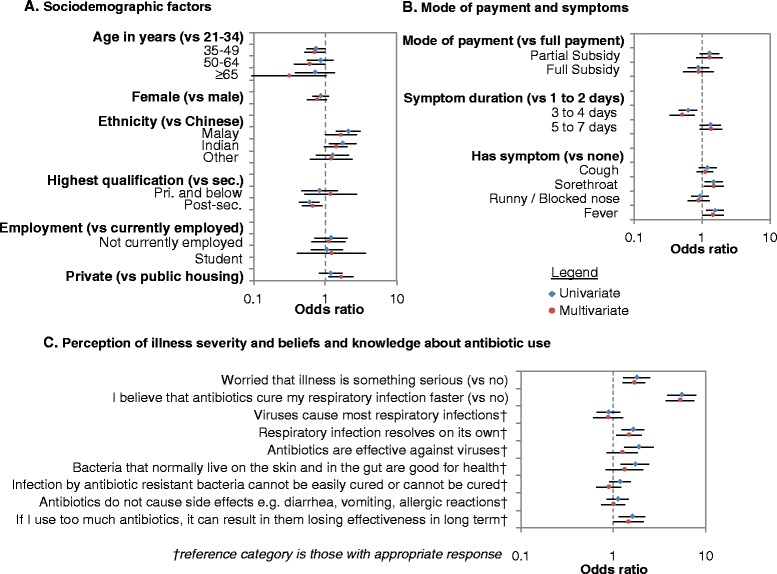
Factors associated with wanting antibiotics on univariate and multivariate analysis. **a** Sociodemographic factors; **b** Mode of payment and symptoms; **c** Perception of illness severity and beliefs and knowledge about antibiotic use. Diamonds give the odds ratio on univariate analysis (*blue*) and multivariate analysis (*red*), with 95 % intervals as error bars

Figure 2 shows factors associated with wanting antibiotics (ORs, 95 % CIs). Indians (1.77, 1.15–2.74) and Malays (2.13, 1.42–3.18) were significantly more likely to want antibiotics than Chinese individuals in the univariate analysis, but the association remained significant only for Malays after adjusting for covariates (1.67, 1.00–2.79, Fig. 2a). Multivariate analysis also suggests that older age groups (vs those aged 21–34, borderline significant) and participants with post-secondary education (0.67, 0.48–0.94, *p* = 0.021 vs secondary education as reference category) were significantly less likely, while those who lived in private housing (vs public housing) were significantly more likely (1.69, 1.13–2.51) to want antibiotics. From Fig. 2b, patients having symptoms lasting 3–4 days (vs 1–2 days, 0.52, 0.34–0.78) were significantly less likely to want antibiotics, while patients having symptoms of sore throat (1.50, 1.07–2.10) or fever (1.46, 1.01–2.12) were significantly more likely to want antibiotics on multivariate analysis. Multiple misconceptions were significantly associated with wanting antibiotics. Not knowing that antibiotics are not effective against viruses (1.91, 1.33–2.74) and that “bacteria that normally live on the skin and in the gut are good for health” (1.75, 1.22–2.51) were significant on univariate but not multivariate analysis (Fig. 2c). However, patients who were worried their illness was serious (1.70, 1.27–2.27), believed that antibiotics cure URTI faster (5.35, 3.76–7.62) and did not know that URTI resolves on its own (2.18, 1.08–2.06) or that using too much antibiotics resulted in their ineffectiveness over the long-term (1.47, 1.00–2.14) were still significantly more likely to want antibiotics on multivariate analysis. Other factors analyzed in Fig. 2 were not significantly associated with wanting antibiotics on neither univariate nor multivariate analysis.

Table 3 presents a stratified analysis by age, gender, ethnicity and education for the belief that antibiotics cure respiratory infection faster, as well as knowledge questions significantly associated on either univariate or multivariate analysis with wanting antibiotics. There was a significant difference among age groups for the questions “Respiratory infection resolves on its own” (*p* = 0.042), and “Bacteria that normally live on the skin and in the gut are good for health” (*p* <0.001), with those ≥65 years old being more likely to respond incorrectly than other age groups. However, this effect was found on bivariate analysis to be related to educational level. There were also statistically significant but modest differences in proportion of males versus females who responded incorrectly to the above two questions, and Malays were significantly more likely not to know that “antibiotics are effective against viruses” (*p* = 0.002); these effects persisted when accounting for educational levels. Patients with lower educational levels, especially the group with primary education and below, were significantly more likely to believe that antibiotics cure respiratory infection faster, as well as have incorrect responses to all four knowledge questions analysed. However, even amongst those with higher education, 63.2 % of the post-secondary group believed that antibiotics cure URTI faster, and 74.5 % did not reject the statement that antibiotics are effective against viruses.

**Table 3:** Sociodemographic variables associated with believing that antibiotics cure respiratory infection faster and incorrect responses to key knowledge questions

Sociodemographic strata		Believe that antibiotics cure respiratory infection faster		Incorrect response to:							
				“Respiratory infection resolves on its own”		“Antibiotics are effective against viruses”		“Bacteria that normally live on the skin and in the gut are good for health”		“If I use too much antibiotics, it can result in them losing effectiveness in the long-term”	
		No. (%)	*p*-value	No. (%)	*p*-value	No. (%)	*p*-value	No. (%)	*p*-value	No. (%)	*p*-value
Age in years	21–34	288 (65.9)	0.804†	203 (46.3)	0.042†	348 (79.5)	0.493†	58 (13.2)	<0.001†	91 (20.8)	0.318†
	35–49	200 (64.9)		150 (48.7)		226 (73.4)		47 (15.3)		55 (17.9)	
	50–64	73 (60.3)		65 (53.7)		101 (83.5)		18 (14.9)		26 (21.5)	
	≥65	33 (71.7)		28 (59.6)		40 (85.1)		22 (46.8)		15 (31.9)	
Gender	Male	285 (64.5)	0.728^*^	200 (45.1)	0.034^*^	359 (81.0)	0.054^*^	84 (19.0)	0.014^*^	96 (21.7)	0.412^*^
	Female	309 (65.7)		246 (52.2)		356 (75.6)		61 (13.0)		91 (19.3)	
Ethnicity	Chinese	388 (61.6)	0.006^*^	297 (47.1)	0.157^*^	486 (77.1)	0.002^*^	94 (14.9)	0.065^*^	118 (18.7)	0.073^*^
	Malay	89 (76.7)		65 (56.0)		105 (90.5)		24 (20.7)		33 (28.4)	
	Indian	69 (71.1)		54 (54.5)		77 (77.8)		21 (21.2)		24 (24.2)	
	Other	48 (69.6)		30 (43.5)		47 (68.1)		6 (8.7)		12 (17.4)	
Highest qualification attained	Pri. or less	35 (74.5)	0.028†	28 (59.6)	<0.001†	45 (95.7)	<0.001	24 (51.1)	<0.001†	20 (42.6)	<0.001†
	Sec.	131 (70.1)		112 (59.9)		163 (87.2)		55 (29.4)		62 (33.2)	
	Post-sec.	428 (63.2)		305 (44.9)		506 (74.5)		66 (9.7)		105 (15.5)	

† *p*-value for Chi-Square test for trend

* *p*-value for Chi-Square test

## Discussion

Majority of our study patients seeking primary health care in Singapore are misinformed about the role of antibiotics in URTI. In particular, poor knowledge was highly prevalent and associated with wanting antibiotics. A third wanted antibiotics, and more than half believed antibiotics could cure their illness faster and had serious misconceptions with regards to the effectiveness of antibiotics for URTI. A substantial minority also had poor and potentially dangerous antibiotic practices such as using left-over antibiotics for themselves and family members. Agreeing that antibiotics cure URTI faster was most strongly associated with wanting antibiotics, and educational level was significantly associated with this belief as well as incorrect knowledge about antibiotics and URTI.

The proportion of our study participants who wanted antibiotics was comparable to those of other studies in Hong Kong and Boston [22, 25], where the proportion who wanted antibiotics ranged from 36 to 39 % respectively, and the proportion with poor antibiotic practices was also comparable to those from a similar study in Malaysia [27]. However, the prevalence of misconceptions in our study was substantially higher than in a similar study in Minnesota [28]; For example, half our study participants did not know that URTI resolves on its own, as compared to 15 % in that study. On the other hand, the prevalence of misconceptions was comparable with a previous local study in Singapore by Tan et al among URTI patients seeking medical care in the public primary care clinics [13]. For example, the belief that antibiotics cure URTI faster was similarly common (61.7 % [Tan et al] vs 65.1 % [our study]). This belief was also most strongly associated with wanting antibiotics, while knowing that viruses caused most respiratory infections was not associated with wanting antibiotics. On the other hand, patients who knew that URTI resolves on its own were significantly less likely to want antibiotics. The content of health education messaging must hence go beyond educating about the causes of URTI to emphasizing its self-limiting nature and the ineffectiveness of antibiotics against viruses.

With regards to presenting symptoms and self-perceived illness severity, we found that patients having sore throat or fever were significantly more likely to want antibiotics. Patients who were worried their illness was something serious were also 1.7 times more likely to want antibiotics, which is comparable to a study in Minnesota [29] where those who rated their symptoms as severe were twice as likely to want antibiotics. Doctors could help address perceptions that fever and sore throat warrant antibiotics, since these could be commonly due to viral infections, as well as reassure worried patients about the benign nature of their illness where appropriate to alleviate unnecessary fears and thus reduce the want for antibiotics.

Regarding sociodemographic factors, younger patients, Malays, and participants living in private housing were more likely to want antibiotics. The reasons are unclear, but may be due to residual confounding from inappropriate beliefs or attitudes that we did not measure or adequately adjust for. Those with higher educational levels were less likely to want antibiotics, similar to findings from a study from Boston [22]. This effect persisted after adjusting for differences in prevalence of misconceptions between educational levels. We did also find a strong association between lower educational levels and higher probability of believing antibiotics cure their respiratory infection faster and having incorrect knowledge on antibiotics, and this finding is consistent with other studies in Malaysia, Korea and Hong Kong [27, 30–32]. Educational level may act through the specific items of knowledge and beliefs covered in our study, as well as other pathways not measured here. Notably, knowledge about antibiotics is not commonly taught during foundation primary schooling years. Less educated participants may also have less access to health education through other information channels, or face language barriers in understanding health education materials. However, given that incorrect knowledge and the belief that antibiotics cure respiratory infections faster is highly prevalent regardless of educational levels, and the link established between these factors and wanting antibiotics, we believe that better public education is important in the fight against inappropriate antibiotic prescriptions in Singapore.

Interventions could take the form of patient education through videos and pamphlets or individualized counseling at primary care clinics, although the efficacy and feasibility of such interventions in Singapore has not been studied and needs to be investigated. Alternatives to clinic-based education would be community-wide public education targeting the entire society; for instance, school-based health education programs have been shown to significantly increase antibiotic-related knowledge among middle-school children in Portugal [33] and Moldova [34]. There are currently no structured health education programs on antibiotic use in the public primary or secondary school syllabus in Singapore. However, the “awareness that bacteria can have both beneficial and harmful effects” has been part of the common lower secondary science syllabus in Singapore [35]; This may be why only 10 % of those who had post-secondary education gave an incorrect response to our question about normal bacterial flora, compared to more than half of those with primary education or less. Therefore, a health education program integrated into the school curriculum which promotes rational use of antibiotics in the community from young may be beneficial. Future research could hence look into the effectiveness of both clinic and community-wide health education programs on proper use of antibiotics.

One limitation we acknowledge was selection bias in our recruitment of GPs. Due to resource constraints, the number of GP clinics in our study was relatively small compared to the total number of GP clinics in Singapore (24 vs 1268 [36]), and recruited from our existing contacts including an academic family medicine network. While it would have been ideal to recruit a more representative sample of GP clinics, another study previously attempted this and encountered dismal response rates [37].

## Conclusion

Our results suggest a substantial proportion of patients in Singapore are misinformed on the role of antibiotics in URTI. Incorrect knowledge about antibiotics, and the belief that antibiotics cure URTI faster was highly prevalent, with the latter being strongly associated with wanting antibiotics. Possible interventions include clinic and community-based education promoting better antibiotic stewardship, although further research is needed to ascertain what type of interventions would be effective.

## Additional files

Additional file 1: Preconsultation Questionnaire for Patients. The pre-consultation questionnaire for patients. Contains a Chinese translation. (DOCX 50 kb)
Additional file 2: Dataset Used. The dataset collected through the surveys. Includes the dataset used in the analysis. (XLSX 317 kb)
Additional file 3: Codebook for dataset supplied. The codebook for interpretation of Material B, the data provided. (XLSX 11 kb)

## Abbreviations

95 % CI: 95 % Confidence interval
GPs: General practitioners
NUS: National University of Singapore
OR: Odds ratio
URTIs: Upper respiratory throat infections
YLLSoM: Yong Loo Lin School of Medicine
