# Supplementary material for: Knowledge, attitudes and practices towards antibiotic use in upper respiratory tract infections among patients seeking primary health care in Singapore
Source: BMC Fam Pract. 2016 Nov 3;17:148. doi: 10.1186/s12875-016-0547-3 (PMC5094024; doi:10.1186/s12875-016-0547-3)
Supplement: Additional file 1: — Preconsultation Questionnaire for Patients. The pre-consultation questionnaire for patients. Contains a Chinese translation. (DOCX 50 kb) [file 12875_2016_547_MOESM1_ESM.docx]

| *<Say this before starting the interview: Your symptoms are likely due to a respiratory infection. I would refer to your current symptoms as common cough and cold in the course of the questionnaire. >*  *《您的症状像是呼吸道感染， 在以下的问答中我将会简称感冒》* | | |
| --- | --- | --- |
| **Part A: Demographics** | | |
| **A0** Study ID |  | |
| **A1** Interviewer name |  | |
| **A2** Date of survey |  | |
| **A3** Patient’s initials |  | |
| **A4** Age |  | |
| **A5** Gender   \| Female \| Male \| \| --- \| --- \| \| F \| M \| | | |
| **A6** Ethnicity ( 种族)   \| Chinese \| Indian \| Malay \| Others \| \| --- \| --- \| --- \| --- \| \| C \| I \| M \| O \| | | |
| **A7** Highest qualification attained *（最高的学历）* | | |
| No Formal Education  *(没有受过任何正式教育)* | | 0 |
| Below Secondary *（小学以下）*  *Primary school or lower* | | 1 |
| Secondary *（中学）*  *O, N level, ITE or similar* | | 2 |
| Post Secondary (Non-Tertiary) *(初级学院或类似）*  *A level, IB program* | | 3 |
| Diploma and Professional Qualification *（专业文凭）*  *Polytechnic, NIE, ITE, SIM, Lasalle, NAFA diploma* | | 4 |
| University *（大学文凭）*  *At least bachelor degree* | | 5 |
| **A8** Employment status | | |
| Unemployed （失业） | | 0 |
| Self-employed （自由职业） | | 1 |
| Employed for wages （ 领薪职位） | | 2 |
| Student （学生） | | 3 |
| Homemaker （家庭主妇） | | 4 |
| Retired （已退休） | | 5 |

| **A9** Housing type（您现在是住在怎么样的房子？） | | | | |
| --- | --- | --- | --- | --- |
| HDB: 1/2 rooms（组屋：一/两房式） | 0 | | | |
| HDB: 3/4 rooms（组屋：三/四房式） | 1 | | | |
| HDB: 5 room/ Executive（组屋：五房式） | 2 | | | |
| Private housing e.g. condominiums, landed properties  （私人住宅： 公寓， 排屋,洋房等） | 3 | | | |
| **A10** Chronic illness （您是否有什么病史？） | | | | |
| Chronic lung diseases e.g. COPD（慢性阻塞性肺病， 抽烟的肺病） | Yes | | No | |
| Asthma （哮喘） | Y | | N | |
| Diabetes （糖尿病） | Y | | N | |
| Hypertension （高血压） | Y | | N | |
| Hyperlipidemia （高胆固醇） | Y | | N | |
| Ischemic heart disease （心脏病） | Y | | N | |
| **A11** Influenza vaccination | | | | |
| Have you ever received vaccination for influenza?  （您打过流感疫苗吗？） | Yes | No | | Don’t know |
| Was your last influenza vaccine within the last 12 months?  （这一年来, 有打过流感疫苗吗？） | Yes | No | | Don’t know |

| *<Say this before starting this part: I am now going to ask you some questions about this current visit >*  *《我现在要问你一些关于这次感冒的问题》* | | | |
| --- | --- | --- | --- |
| **Part B: Current visit** | | | |
| **B1** How many days were you sick before you came to seek consultation? (Cross the day of consultation and circle the day the patient first felt unwell) （你这一次病了多久才来看医生？） | | | |
| Mon Tues Wed Thurs Fri Sat Sun Mon Tues Wed Thurs Fri Sat Sun Mon Tues Wed Thurs Wed Thurs Fri Sat Sun | | | |
| **B2** Why are you seeing the doctor? （你看医生的原因是否包括以下的因素：） | | | |
| To get Mc （为了请病假，留在家休息） | Yes | No | |
| To get medication （为了拿药） | Y | N | |
| Worried that you are suffering from something serious  （因为担心自己病得很严重） | Y | N | |
| You are worried that if not treated early, your respiratory infection will result in complications  （因为担心若不看医生，自己的病情会有并发症） | Y | N | |
| You just need reassurance from your doctor  （因为您希望能得到医生的安慰，确保自己没事） | Y | N | |
| The respiratory infection is lasting longer than expected  （因为您的感冒比预料中更长） | Y | N | |
| **B3** What did you do prior to this visit? (您来看医生之前，是否接受过任何的治疗方式?) | | | |
| Traditional measures  *Drinking more water, covering more blankets to sweat it out*  （传统方式（例如：多喝水，多盖被流汗）) | Yes | | No |
| Traditional medication  （服用传统药物 （例如：中药）） | Y | | N |
| Over-the-counter medication  （服用从西药房购买的药物） | Y | | N |
| Prescribed medication  （服用其他医生开的药） | Y | | N |
| I went to visit another doctor  （看过另外一位医生） | Y | | N |
| Others: （其他原因） | Specify: | | |
| **B4** How are you paying for your consultation? （您今天将如何付款？） | | | |
| Fully out of your pocket  （自己付现金） | 0 | | |
| Subsidized *Government schemes e.g. CHAS*  （政府津贴（例如：保健援助卡CHAS, 建国一代配套）） | 1 | | |
| Free *Insurance schemes*  （免费（例如：私人或公司保险）） | 2 | | |

| *<Say this before starting this part: I am now going to ask you some general questions about what you do when you are down with common cough and cold in the past>*  *《我现在要问你一些关于平时感冒时候的问题》* | | | | |
| --- | --- | --- | --- | --- |
| **Part C: Attitudes** | | | | |
|  | Strongly disagree | Disagree | Agree | Strongly agree |
| **C1** Self-medication is the first thing I do when I have a respiratory infection.  （感冒时, 我会先自己服用药物） | 1 | 2 | 3 | 4 |
| **C2** My respiratory infection recovers faster when I see a doctor.  （ 我觉得看医生会让我的感冒康复的更快。） | 1 | 2 | 3 | 4 |
| **C3** I believe that antibiotics cure my respiratory infection faster.  （我觉得服用抗生素能使我的感冒更快地康复。） | 1 | 2 | 3 | 4 |
| **C4** I will change my doctor because according to my opinion they do not prescribe antibiotics enough.  （如果我觉得医生没开足够的抗生素,我会去看另一位医生。） | 1 | 2 | 3 | 4 |
| **C5** I know which medicines are antibiotics when I take cold medicines.  （在服用感冒药时，我能辨认出哪一种药是抗生素。） | 1 | 2 | 3 | 4 |
| **C6**I take left-over antibiotics when I have similar respiratory infection symptoms.  （当我有类似感冒的症状时，我会服用家里之前剩下的抗生素。） | 1 | 2 | 3 | 4 |
| **C7** I normally keep antibiotic stock at home in case of emergency.  (我平常会在家里存放抗生素，以便有需要时服用) | 1 | 2 | 3 | 4 |
| **C8** If my family is sick, I usually give my antibiotic to them.  (我的家人若是生病了，我会给他们吃我的抗生素) | 1 | 2 | 3 | 4 |

| **Part D: Wishes and expectations** | | | | |
| --- | --- | --- | --- | --- |
|  | Strongly disagree | Disagree | Agree | Strongly agree |
| **D1** I want to receive antibiotics  (我想拿到抗生素) | 1 | 2 | 3 | 4 |
| **D2** I want other medication for my respiratory infection  *Antipyretics, antihistamine, cough medicine etc*  *(*我想得到其他感冒药，比如退烧药，流鼻涕药， 止咳药等) | 1 | 2 | 3 | 4 |
| *<Do the following questions only if patients indicate that they want to receive antibiotics>* | | | | |
| **D3** I will accept the doctor’s decision to not prescribe antibiotics if he explains to me why  (如果医生没给抗生素，只要他跟我解释为什么，我会接受他的决定) | 1 | 2 | 3 | 4 |
| **D4** I will ask the doctor for antibiotics if not offered  (如果医生没有给抗生素，我会问他要) | 1 | 2 | 3 | 4 |
| **D5** I will go to another doctor if antibiotics are not prescribed  (如果没有得到抗生素，我会去看别的医生) | 1 | 2 | 3 | 4 |

| **Part E: Knowledge about antibiotic use** | | | |
| --- | --- | --- | --- |
|  | Yes | No | Not sure |
| **URTI** | | | |
| **E1** Viruses cause most respiratory infections. (大多数感冒是因为病毒感染) | 1 | 2 | 3 |
| **E2** What else do you think cause respiratory infections? (您认为以下的哪些因素会导致呼吸道感染？) | | | |
| Environment eg. stress ( 环境 , 例如：压力) | 1 | 2 | 3 |
| Heatiness (热气,上火) | 1 | 2 | 3 |
| Weather (天气) | 1 | 2 | 3 |
| Germs (病菌) | 1 | 2 | 3 |
| Others (其他): | Specify: | | |
| **E3** Respiratory infection resolves on its own.  （即使不吃药,感冒也会自然地康复） | 1 | 2 | 3 |
| **E4** Without taking antibiotics, how long do you think your respiratory infection takes to get better?  (若您不服用抗生素, 您认为自己的呼吸道感染会持续多久之后才恢复) | | | |
| 1-2 days （一至两天） | 1 |  |  |
| 3-5 days （三至五天） | 1 |  |  |
| 5-7 days （五至七天） | 1 |  |  |
| >7 days （七天以上） | 1 |  |  |
| **Role of antibiotics (抗生素的作用)** | | | |
| **E5** Antibiotics are effective against viruses. (抗生素对抗病毒有效) | 1 | 2 | 3 |
| **E6** Antibiotics are effective against bacteria. (抗生素对抗细菌有效) | 1 | 2 | 3 |
| **E7** When do you think antibiotics are needed? (您认为以下哪一种症状需要服用抗生素？) | | | |
| Fever (发烧) | 1 | 2 | 3 |
| Chesty cough (咳嗽) | 1 | 2 | 3 |
| Sore throat (喉咙痛) | 1 | 2 | 3 |
| Runny nose with yellowish/greenish mucus (黄/绿色的鼻涕) | 1 | 2 | 3 |
| Yellowish/greenish phlegm (黄/绿色的痰) | 1 | 2 | 3 |
| Others (其他): | Specify: | | |
| **Anti-microbial resistance (有关抗药性的细菌)** | | | |
| **E8** We have good bacteria that normally live in us e.g. skin and gut. （我们的身体上常有一些对身体有益的细菌） | 1 | 2 | 3 |
| **E9** Antibiotics do not kill these bacteria.  （抗生素不会消灭这些身体上‘好’的细菌） | 1 | 2 | 3 |
| **E10** Antibiotic resistance means that antibiotics will not kill bacteria.  （抗药性细菌是指一些不会被抗生素消灭的细菌） | 1 | 2 | 3 |
| **E11** Infection by antibiotic resistant bacteria cannot be easily cured  （若被抗药性細菌感染，病情便不容易治好） | 1 | 2 | 3 |
| **E12** Scientists can continuously produce new antibiotics that can kill resistant bacteria. （科学家可以不断地制造新的抗生素来消灭这些抗药性細菌） | 1 | 2 | 3 |
| **Adverse effects（抗生素的副作用）** | | | |
| **E13** Antibiotics do not cause side effects e.g. diarrhea, vomiting, allergic reactions （抗生素不会导致副作用如： 泻肚子，呕吐，过敏反应） | 1 | 2 | 3 |
| **E14** If I use too much antibiotics, it can result in them losing effectiveness in long term. （若过度使用抗生素，将导致它在长期内失去效用） | 1 | 2 | 3 |
| **Administration of antibiotics （抗生素的服用）** | | | |
| **E15** It is okay to stop taking an antibiotic after symptoms are gone.  （若感冒有好转的现象,我们可以停止服用抗生素） | 1 | 2 | 3 |
| **E16** Taking lesser antibiotics than prescribed is healthier than taking the full course prescribed.  （服用少于医生所开的抗生素药量比把全部抗生素吃完健康） | 1 | 2 | 3 |
